# Functional connectivity in multiple sclerosis modelled as connectome stability: A 5-year follow-up study

**DOI:** 10.1177/13524585211030212

**Published:** 2021-07-14

**Authors:** Einar August Høgestøl, Samuele Ghezzo, Gro Owren Nygaard, Thomas Espeseth, Piotr Sowa, Mona K Beyer, Hanne Flinstad Harbo, Lars T Westlye, Hanneke E Hulst, Dag Alnæs

**Affiliations:** “Department of Neurology, Neuroscience Research Unit, Multiple Sclerosis Research Group University of Oslo & Oslo University Hospital; Institute of Clinical Medicine, University of Oslo, Oslo, Norway; NORMENT, Division of Mental Health and Addiction, Oslo University Hospital, Oslo, Norway/Department of Neuroscience, Vrije Universiteit Amsterdam, Amsterdam, The Netherlands; Department of Neurology, Neuroscience Research Unit, Multiple Sclerosis Research Group University of Oslo & Oslo University Hospital, Oslo, Norway; Department of Psychology, University of Oslo, Oslo, Norway; Bjørknes College, Oslo, Norway; Division of Radiology and Nuclear Medicine, Oslo University Hospital, Oslo, Norway; Institute of Clinical Medicine, University of Oslo, Oslo, Norway/Division of Radiology and Nuclear Medicine, Oslo University Hospital, Oslo, Norway; “Department of Neurology, Neuroscience Research Unit, Multiple Sclerosis Research Group University of Oslo & Oslo University Hospital; Institute of Clinical Medicine, University of Oslo, Oslo, Norway; NORMENT, Division of Mental Health and Addiction, Oslo University Hospital, Oslo, Norway/Department of Psychology, University of Oslo, Oslo, Norway/KG Jebsen Centre for Neurodevelopmental Disorders, University of Oslo, Oslo, Norway; Department of Neuroscience, Vrije Universiteit Amsterdam, Amsterdam, The Netherlands/Department of Anatomy and Neurosciences, MS Center Amsterdam, Amsterdam Neuroscience, Amsterdam UMC, Amsterdam, The Netherlands; NORMENT, Division of Mental Health and Addiction, Oslo University Hospital, Oslo, Norway; Bjørknes College, Oslo, Norway

**Keywords:** Multiple sclerosis, neuroimaging, functional neuroimaging, connectome, cohort studies, longitudinal studies, neuropsychological tests

## Abstract

**Background:**

Brain functional connectivity (FC) in multiple sclerosis (MS) is abnormal compared to healthy controls (HCs). More longitudinal studies in MS are needed to evaluate whether FC stability is clinically relevant.

**Objective:**

To compare functional magnetic resonance imaging (fMRI)-based FC between MS and HC, and to determine the relationship between longitudinal FC changes and structural brain damage, cognitive performance and physical disability.

**Methods:**

T1-weighted MPRAGE and resting-state fMRI (1.5T) were acquired from 70 relapsing-remitting MS patients and 94 matched HC at baseline (mean months since diagnosis 14.0 ± 11) and from 60 MS patients after 5 years. Independent component analysis and network modelling were used to measure longitudinal FC stability and cross-sectional comparisons with HC. Linear mixed models, adjusted for age and sex, were used to calculate correlations.

**Results:**

At baseline, patients with MS showed FC abnormalities both within networks and in single connections compared to HC. Longitudinal analyses revealed functional stability and no significant relationships with clinical disability, cognitive performance, lesion or brain volume.

**Conclusion:**

FC abnormalities occur already at the first decade of MS, yet we found no relevant clinical correlations for these network deviations. Future large-scale longitudinal fMRI studies across a range of MS subtypes and outcomes are required.

## Introduction

Multiple sclerosis (MS) is a disease characterized radiologically by the accumulation of lesions in white and grey matter over time throughout the central nervous system (CNS).^1,2^ The white matter of the brain constitutes a framework for structural connectivity between brain regions, supporting large-scale brain functional network connectivity,^3–5^ collectively termed the functional connectome. Accumulating evidence has demonstrated abnormal patterns of brain functional connectivity (FC) in MS patients as compared to healthy controls (HCs).^4,6–12^ While extensive evidence shows that FC abnormalities are associated with clinical disability in MS,^4,6–10,12^ there is a complex pattern of increased and decreased connectivity, both between brain regions directly affected by lesions, as well as putative secondary cascade effects in distal brain regions.^4^ In addition, the heterogeneity across patients in lesions location is likely to further contribute to individual differences in FC aberrations in MS.^4,13^

The complex interplay between FC dysregulation and clinical impairment cannot be reduced to the effects of local FC increase or decrease in a cross-sectional setting.^4,13^ A longitudinal, individual-based and connectome-wide approach that accounts for FC stability following structural damage is warranted to better understand the complex interplay between brain lesions, disease progression and heterogeneous FC aberrations in MS.^4,13–15^ This can be conceptualized as connectome stability, where the instability could refer to both compensatory ‘good’ changes, and aberrant connectivity caused by lesions giving rise to disorganization in FC (like maladaptation).^16^

Here, we investigated a prospectively collected MS cohort with comprehensive imaging and clinical data over 5 years. First, we compared baseline resting-state functional magnetic resonance imaging (rs-fMRI) in MS patients to a group of age and sex-matched HC. Second, we used longitudinal rs-fMRI data from the patients to compute regional and global indices of longitudinal connectome stability.^16^ This measure reduces complex connectome-wide changes into a single, individual-level marker of longitudinal FC stability. We investigated the clinical relevance of the FC-stability measure through linear associations with disease progression, brain volume, lesion load, and clinical and cognitive outcomes. Our hypotheses were (1) MS affects fMRI-based FC in the first decade of disease, as compared with HC, (2) FC stability over a 5-year interval is related to fewer structural changes in patients and (3) FC stability is related to better cognitive and physical disability in MS.

## Material and methods

### Participants

The 76 MS patients were part of a prospective longitudinal MS study at the Oslo University Hospital.^17^ All patients were diagnosed between January 2009 and October 2012 with relapsing-remitting multiple sclerosis (RRMS),^18^ with one patient later re-evaluated to be primary-progressive (PP) multiple sclerosis. Five years after baseline, 62 patients were re-examined. Six and two MS patients were not examined with rs-fMRI sequence at baseline and follow-up, respectively. For all the inclusion criteria, please refer to Supplemental Data.

We included cross-sectional data from 94 age and sex-matched HC participants from the Norwegian Cognitive NeuroGenetics (NCNG) cohort, recruited through newspaper advertisements.^19^ Inclusion criteria were age between 20 and 80 years, no previous diseases affecting the CNS, no previous psychiatric disorders and no previous or current substance abuse.

The project was approved by the regional ethical committee of South Eastern Norway (REC ID: 2011/1846, 2016/102 and 2009/2070), and all participants received oral and written information and gave their written informed consent.

### Neurological and cognitive assessment

All MS patients completed a comprehensive neurological examination at baseline and follow-up, including assessment of the Expanded Disability Status Scale (EDSS), Timed 25-Foot Walk (T25-FW) and the nine-hole peg test (9HPT) within the same week as the MRI scan. No evidence of disease activity (NEDA) was defined as the absence of clinical relapses, new or enlarging MRI lesions, new gadolinium (Gd)-enhancing lesions and EDSS progression.

Furthermore, all participants completed a comprehensive cognitive evaluation with BICAMS^20^ and some additional tests (Supplementary Table 1), evaluating the cognitive domains of: processing speed, executive functioning, visuospatial and verbal memory. To minimize practice-related effects, we used validated subtests whenever possible. For cognitive tests for HC, please refer to Supplemental Data.

In order to obtain measures of cognition, we first calculated *Z*-scores for each of the tests administered using the average performance, and standard deviation, of matched HC at baseline as a reference. We then grouped the *Z*-scores into the four domains previously described and averaged them within each domain, obtaining a domain-specific measure of cognition at the patient level. Finally, we averaged the scores across the four domains, obtaining a measure of overall cognition for each MS patient at both time points. Similarly, we averaged the *Z*-scores for T25-FW and 9HPT to obtain a measure of physical ability for the MS subjects, both at baseline and follow-up. Since measures of physical ability for HC were missing, we used the average performance, and standard deviation, of the patient cohort itself as reference to calculate the *Z*-scores for physical ability.

### MRI acquisition and structural MRI pre- and post-processing

All MS and HC participants were scanned using the same 1.5T scanner (Avanto; Siemens Medical Solutions, Erlangen, Germany) equipped with a 12-channel head coil, with the same rs-fMRI sequence and parameters for both time points (Supplemental Data). A harmonized pre- and post-processing pipeline was used for all structural (T1 weighted and MPRAGE) and rs-fMRI data to minimize confounding effects (Supplemental Data).

### ICA and FC matrices

The cleaned and Montreal Neurological Institute (MNI)-conformed rs-fMRI data were submitted to temporal concatenation group independent component analysis (gICA) using MELODIC^21^ with a model order of 40 independent components (ICs).^22–25^ These group-level spatial components were then used as spatial regressors against each participant’s rs-fMRI data set to estimate subject-specific component spatial maps and associated time series (dual regression).^22,24–26^ After removing 15 ICs classified as non-CNS based on visual assessment and correlation with the Smith networks (Supplementary Figure 1), we extracted a total of 25 ICs for further analysis (Supplementary Figure 2). The times series of the noise-ICs was regressed out of the time series of the kept ICs. We calculated connectivity matrices using full as well as regularized partial correlations with automatic estimation of regularization parameters at the individual level.^27,28^ Based on the Euclidean distances of the full temporal correlations, the ICs grouped into four clusters largely representing (1) and (2) default mode network (DMN) and frontoparietal areas, (3) auditory network and (4) sensory/motor areas (Supplementary Figure 3 and Supplementary Table 1).^22–24^ Since partial correlations are assumed to represent direct connections between nodes, these were used in further analyses.

### Functional connectome stability

We computed an index of each MS patient’s longitudinal brain functional connectome stability using the following procedure: for each patient and for each time point, we extracted the node–node connectivity measures from the whole-brain connectivity matrix, creating a vector of length 300 (25 ICs and 300 unique links between them). Then, we calculated the within-subject Spearman correlation coefficient between the baseline and follow-up connectivity values, in this way obtaining a measure of whole-brain functional connectome stability.^16^ Similarly, and to complement the full-brain index, we computed within-network connectome stability for each of the four identified network-clusters. This measure captures any change in the rank of edges between baseline and follow-up, independent of their direction, thus providing individual-level global and network-specific measures of longitudinal FC stability for the MS patients. To further validate the implementation of the stability index as a global measure of FC changes over time, we correlated functional connectome stability with the sum of the squared differences in FC between baseline and follow-up for all edges in each of the MS patients.

### Statistical analysis

For statistical analyses, we used R^29^ and MATLAB version 2014a (MathWorks Inc., Natick, MA, USA, 2018). Group-level changes in performance between baseline and follow-up were tested by paired sample *t*-tests. We used separate multiple linear regressions to test for differences in whole-brain FC stability, within-network FC stability, and FC at the level of single edges between HC and MS patients at baseline. We performed paired sample *t*-tests to assess edge- and network-wise changes in FC over time in the MS cohort.

To test for associations between disease progression and functional abnormalities, we used multivariate linear models to compare whole-brain, and within-network, connectome stability between patients with and without evidence of disease activity (EDA). To test the relation between structural damage and FC abnormalities, we correlated the connectome stability index with measures of lesion-filled brain volume and lesion load at baseline, and with brain atrophy and lesion change at follow-up. For lesion volumes, we used log transformation to account for the lack of normal distribution in the resulting volumes.

Finally, we used multiple linear regressions to assess the relationship between connectome stability and cognition and physical ability at follow-up, and the change in these tests between baseline and follow-up. We adjusted for sex, age, estimated mean relative motion and temporal signal-to-noise ratio (tSNR) in all linear models. Significance was defined as *p* < 0.05 (corrected). We used false discovery rate (FDR; *p* < 0.05) for the case–control comparisons, the longitudinal analyses and for models evaluating the clinical relevance of connectome stability. We applied permutation testing when comparing connectome stability between EDA and NEDA patients to derive exact *p*-values, since this approach does not rely on any assumption about the distribution of the stability measure. Specifically, we obtained an empirical null-distribution of estimates for the group difference across 1000 permutations randomly permuting the group-label. The family-wise error was controlled by collecting the maximum test statistic across the whole-brain and within-network tests for each permutation.^30^ The resulting *p*-value was calculated by dividing the number of permuted beta-values equal or larger than the point estimate by the total number of permutations.

## Results

### Sample characteristics

The MS sample included 71% (*n* = 54) females. At follow-up, 44% (*n* = 27) of the patients met criteria for NEDA. The median EDSS (2.0) score did not change after 5 years. One patient developed secondary-progressive multiple sclerosis (SPMS) at follow-up. Mean time between baseline and follow-up was 4.5 years (standard deviation (SD) = 0.4 years, range = 3.7–5.4 years). Disease-modifying treatment (DMT) was used by 78% and 69% of the patients at baseline and follow-up, respectively (Table 1). A more detailed description on the differences between NEDA and EDA patients can be found in Supplementary Table 2. The NCNG cohort was matched to the MS sample at baseline by age (mean years = 34.89, SD = 9.17) and sex (74% female). The Amsterdam HC sample were mostly female (70% female, *n* = 222, mean years = 41.86, SD = 11.44).

**Table 1.** Demographic and clinical characteristics of the multiple sclerosis patients.

(a) Demographic characteristics	Baseline	Follow-up
	*n* = 76	*n* = 62
Female, *n* (%)	54 (71)	44 (71)
Age, mean years (SD)	35.3 (7.3)	40.5 (7.2)
Disease duration, mean months (SD)	71.7 (63)	125.1 (60.2)
Age at first symptom, mean years (SD)	29.3 (6.7)	
Months since diagnosis, mean (SD)	14.0 (11.9)	66.4 (14.5)
*Disease-modifying treatment*		
None, *n* (%)	17 (22)	19 (31)
First-line treatment, *n* (%)	49 (65)	23 (37)
Second-line treatment, *n* (%)	10 (13)	20 (32)
(b) Clinical evaluation		
*Multiple sclerosis classification*		
RRMS, *n* (%)	75 (99)	60 (96)
PPMS, *n* (%)	1 (1)	1 (2)
SPMS, *n* (%)		1 (2)
*Neurological disability*		
EDSS, median (SD, range)	2.0 (0.9, 0–6)	2.0 (1.3, 0–6)
MSSS (SD)	4.9 (1.9)	2.6 (1.8)
Number of total relapses, mean (SD)	1.8 (1)	2.6 (1.3)
FSS, mean (SD)	4.2 (1.7)	4.1 (1.9)
(c) NEDA assessment		
NEDA-3, *n* (%)		27 (44)

SD: standard deviation; RRMS: relapsing-remitting multiple sclerosis; PPMS: primary-progressive multiple sclerosis; SPMS: secondary-progressive multiple sclerosis; EDSS: Expanded Disability Status Scale; MSSS: Multiple Sclerosis Severity Scale; FSS: Fatigue Severity Scale; NEDA: no evidence of disease activity.

At the group level, MS patients improved on Color Word Interference Test (CWIT) and California Verbal Learning Test-II (CVLT-II) over time, while no significant differences between baseline and follow-up were identified for Symbol Digit Modalities Test (SDMT), T25-FT and 9HPT (Supplementary Table 3). Only two participants displayed a significant decrease in physical ability, and none in average cognition (Supplementary Figures 4 and 5). We found no associations between rs-fMRI signal-to-noise ratio and mean relative motion and clinical and cognitive outcomes (Supplementary Table 4).

### FC abnormalities in MS versus HCs at baseline

**Figure 1.**
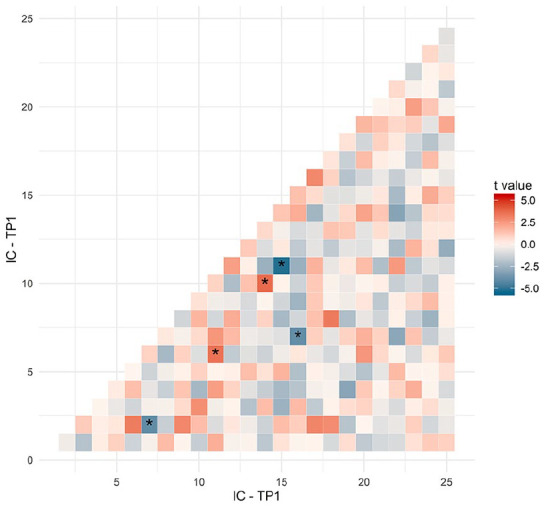
Edge-wise analyse of functional connectivity (FC) abnormalities. *T*-values from multivariate linear regressions assessing differences in FC at the level of single connections between MS and HCs. Red colours indicate the increased FC in MS, and blue colours indicate a decrease in FC. *p-value significant after correction for multiple testing by false discovery rate (*q* = 0.05).

Figure 1 and Supplementary Figure 6 show the results from the edge-wise comparisons in FC between MS and HCs, revealing edges with both increased as well as decreased connectivity in patients at baseline. FC in DMN and frontoparietal networks (networks 1 and 2) was significantly different from HC (Table 2). Edge-wise analysis showed that a connection (IC11–IC15) between nodes belonging to DMN and frontoparietal networks (network 1) was weaker in MS relative to controls (β = −0.1, *t*(135) = −5.21, *p* = −0.0002), while another edge (IC6–IC11) within the same networks was stronger in patients compared to HC (β = 0.07, *t*(135) = 3.54, *p* = 0.032). An edge (IC10–IC14) between nodes belonging to DMN and frontoparietal networks (network 2) was higher in MS (β = 0.08, *t*(135) = 3.74, *p* = 0.002) and another, (IC7–IC16), between nodes belonging to the auditory networks (network 3) was weaker in MS patients relative to HC (β = −0.08, *t*(135) = −3.94, *p* = 0.001). Also, a connection (IC2–IC7) between one node belonging to auditory networks (network 3) and one node belonging to sensory and motor networks (network 4) was weaker in MS patients compared to HC (β = −0.08, *t*(135) = −4.02, *p* = 0.001).

**Table 2.** Within network functional connectivity abnormalities in MS.

	Beta coefficient	*T*-value	Standard deviation	*p*-value
Full brain	−0.001	−1.28	0.001	0.2
Network 1 DMN and frontoparietal nodes	−0.017	−3.56	0.005	0.002*
Network 2 DMN and frontoparietal nodes	−0.011	−3.14	0.003	0.004*
Network 3 Auditory nodes	−0.009	−1.59	0.006	0.15
Network 4 Sensory and motor nodes	0.002	0.41	0.005	0.68

MS: multiple sclerosis; DMN: default mode network.

Results of multivariate linear regression models corrected for sex, age, mean relative motion and signal-to-noise ratio. *p*-values corrected for multiple testing by false discovery rate.

* *p*-value significant after correction for multiple testing by false discovery rate.

At the group level, paired sample *t*-tests revealed no significant edge-wise longitudinal FC changes in the MS cohort (Supplementary Figures 7 and 8 and Supplementary Table 5). Additional analyses to evaluate the robustness of our approach, including careful lesion masking during the estimation of the node time series, excluding the two subjects with progressive forms of MS, or expanding the IC model order to 50, did not change the main effects or interpretation of the results (Supplementary Table 8 and Supplementary Figure 9).

### Longitudinal functional connectome stability

**Figure 2.**
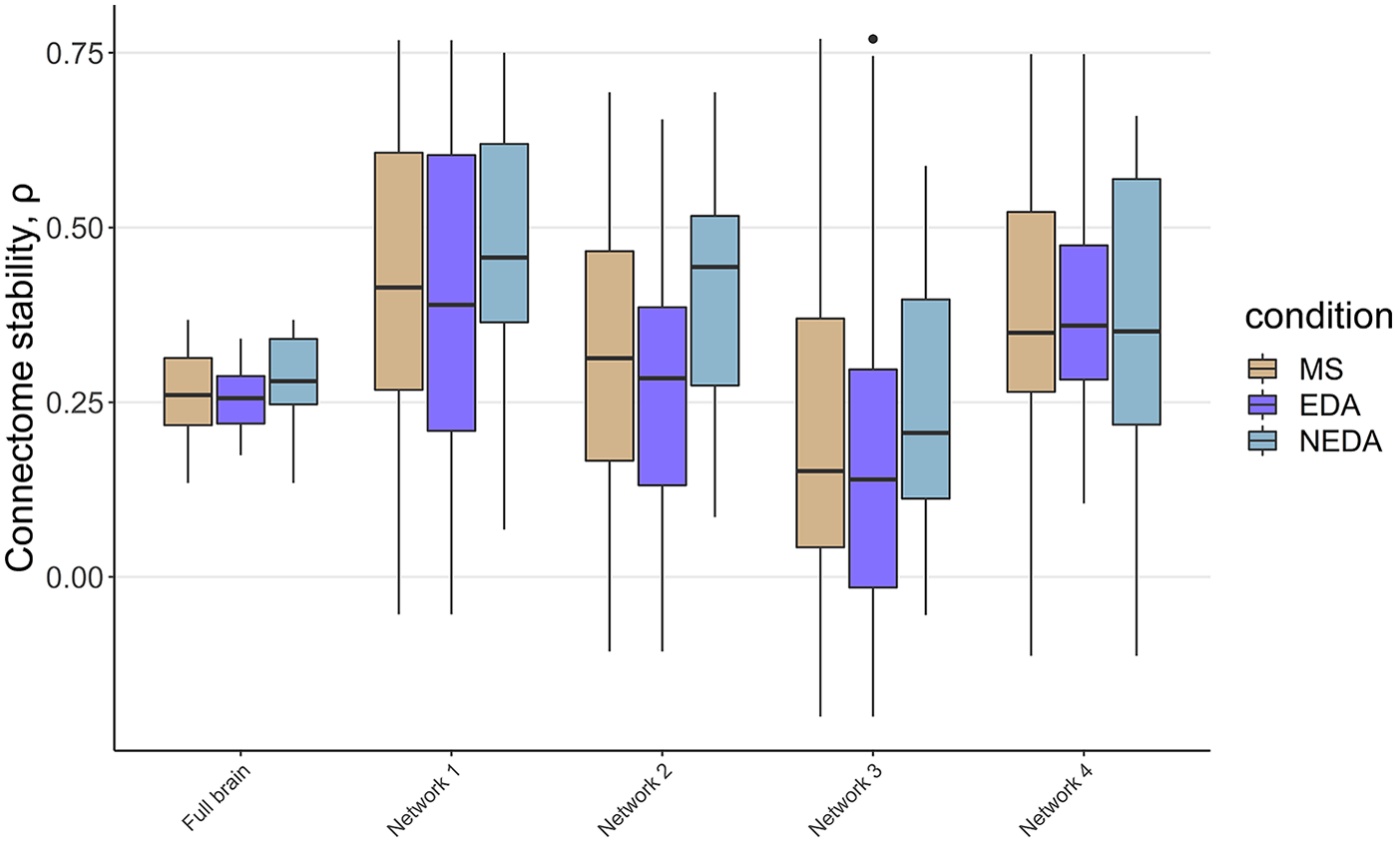
Stability of the brain functional connectome between baseline and follow-up for the MS sample as a whole, and for the subgroups of EDA and NEDA, respectively, for the global estimate and all resulting networks.

The stability of the brain functional connectome in the whole MS cohort, and in the EDA and NEDA subgroups is depicted in Figure 2, enabling visualization of FC changes due to disease progression. Functional connectome stability of the nodes clustering with DMN and frontoparietal networks (network 2) was nominally lower in EDA patients compared to NEDA patients (β = 0.14, *t*(34) = 2.26, *p* = 0.03), but this effect did not survive correction for multiple testing (Supplementary Table 6). We found no association between functional connectome stability and measures of lesion-filled (Supplementary Table 7) brain volume and lesion load at baseline (Supplementary Figure 10), nor with brain atrophy or lesion volume changes (Supplementary Figure 11).

Functional connectome stability was highly correlated with the sum of the squared differences in FC between baseline and follow-up (ρ = −0.59, *p* < 0.0001).

### Clinical relevance of FC changes

**Figure 3.**
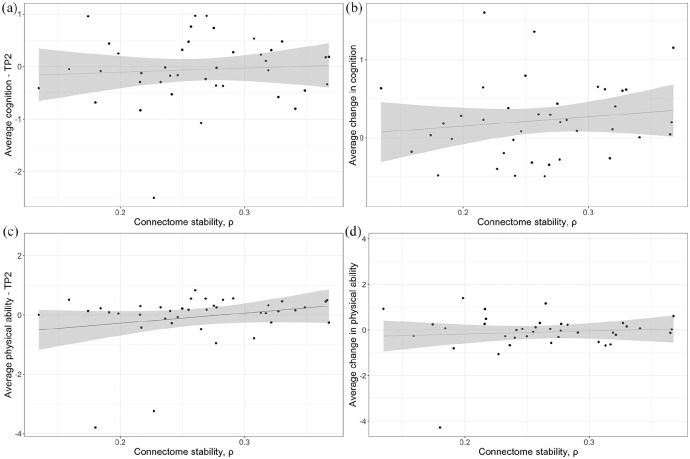
Effect of functional connectome stability on cognitive performance and physical ability. (a) Effect of FC stability on average cognition at follow-up, β = 1.98, *t*(34) = 1.20, *p* = 0.54. (b) Effect of FC stability on average change in cognition, β = 1.00, *t*(33) = 0.64, *p* = 0.69. (c) Effect of FC stability on physical ability at follow-up, β = 4.56, *t*(34) = 2.00, *p* = 0.21. (d) Effect of FC stability on average change in physical ability, β = 0.89, *t*(33) = 0.33, *p* = 0.75. *p*-values corrected for multiple testing by false discovery rate.

Finally, we tested for associations between changes in FC and cognitive performance and physical ability using general linear models with average cognition and physical ability at follow-up as dependent variables, covarying for age, sex, signal-to-noise ratio and mean relative motion. Younger age (β = −0.03, *t*(34) = −2.08, *p* = 0.045) was associated with better cognitive performance at follow-up (Figure 3(a)), but did not survive correction for multiple testing (adjusted *p* = 0.089). No significant associations between longitudinal changes in cognitive performance and functional connectome stability, age, sex, signal-to-noise ratio nor mean relative motion were found. (Figure 3(b)).

For physical ability, lower functional connectome stability (β = 4.56, *t*(34) = 2.00, *p* = 0.05), higher age at follow-up (β = −0.05, *t*(34) = −2.45, *p* = 0.02) and sex, with women scoring better than men, (β = 0.70, *t*(34) = 2.46, *p* = 0.02) were nominally associated with decreased physical ability at follow-up (Figure 3(c)), but none of these effects survived correction for multiple testing. Neither stability of the brain functional connectome, age, sex, signal-to-noise ratio nor mean relative motion were associated with changes in physical performance over time (Figure 3(d)).

## Discussion

In this 5-year longitudinal prospective MS study, we investigated the clinical relevance of an fMRI-derived individual-level longitudinal index of global functional connectome stability. In addition, we performed a cross-sectional case–control comparison with a matched HC sample, assessing aberrant FC in MS patients at baseline.

The case–control comparison replicated previous reports of FC differences in MS compared to HC,^4,11,14^ supporting our first hypothesis that FC aberrations are present already in the first decade of acquiring MS. Schoonheim et al.^31^ (2010) proposed a model for functional reorganization of the brain in relation to structural damage and clinical impairment in MS, in which, at least in the first decade of the disease, a compensatory increase in FC buffers clinical and cognitive consequences of MS-related structural damage. Accumulating evidence has since been established, describing a more complex pattern of FC aberrations in MS.^4,11,13^

Investigating the longitudinal stability of the brain functional connectome at the individual level allowed us to study the complex and heterogeneous dynamics of FC aberrations in MS. The concept of connectome stability enabled us to test the hypothesis presented by Schoonheim et al.,^31^ accounting for the whole set of FC alterations characterizing MS. Our analysis revealed that connectome stability of nodes clustering with the DMN and frontoparietal networks (network 2) were nominally lower in EDA patients compared to NEDA patients; however, the result did not remain significant after correcting for multiple testing. Furthermore, neither lesion-filled brain volume nor lesion load was associated with connectome stability in both cross-sectional and longitudinal models (Supplementary Figures 10 and 11). Importantly, we found no significant associations between connectome stability and progression of cognitive and physical impairment. A possible explanation for the lack of clinical associations might be that our subjects were remarkably stable at follow-up (Supplementary Figures 4 and 5). At follow-up, 44% of the MS subjects were classified with NEDA (Supplementary Table 2)

Similar implementations of connectome stability have previously been used to study mental health in youth,^32^ severe mental disorders^16^ and cognitive ageing.^33^ In order to supplement the rank-based estimate, we correlated the index of connectome stability with an alternative operationalization based on the sum of the squared differences between time points, which revealed highly corresponding estimates. In general, our results are in line with the few previous findings showing FC abnormalities in the first decade of the disease and, in accordance with Rocca et al., we found that FC alterations did not correlate with lesion load.^4,14^

Limitations of this study include lack of MRI follow-up for HC, preventing this study to draw conclusions on whether the longitudinal changes are specific to MS. Since HC only performed cognitive tests at baseline, we used these results to create the *Z*-scores for MS patients at follow-up. *Z*-scores for physical ability were based on analyses of performance of MS patients only. To reduce the practice effects on the cognitive tests, the MS patients completed alternative subtests in the cognitive test battery to reduce the task familiarity effects.^34^ The sample size of our MS cohort is comparable with that of previous studies investigating FC longitudinally, but larger samples may be needed to identify subtle associations between brain network dynamics and clinical characteristics.^14^

## Conclusion

In this longitudinal study, we found that our MS cohort was clinically stable with preserved cognitive abilities. We revealed FC abnormalities at baseline supporting earlier studies showing FC aberrations already in the first decade of MS. We found that connectome-wise FC stability cannot be predicted by the level of lesion load or brain volume, both in a cross-sectional and longitudinal setting. Future large-scale longitudinal fMRI studies are needed to confirm the sensitivity and clinical relevance of the fMRI-derived connectome stability index, and to map associations with trajectories of physical and cognitive changes in MS, preferably also with longitudinal HC fMRI data.

## Supplemental Material

sj-docx-1-msj-10.1177_13524585211030212 – Supplemental material for Functional connectivity in multiple sclerosis modelled as connectome stability: A 5-year follow-up study Click here for additional data file. Supplemental material, sj-docx-1-msj-10.1177_13524585211030212 for Functional connectivity in multiple sclerosis modelled as connectome stability: A 5-year follow-up study by Einar August Høgestøl, Samuele Ghezzo, Gro Owren Nygaard, Thomas Espeseth, Piotr Sowa, Mona K Beyer, Hanne Flinstad Harbo, Lars T Westlye, Hanneke E Hulst and Dag Alnæs in Multiple Sclerosis Journal

sj-docx-2-msj-10.1177_13524585211030212 – Supplemental material for Functional connectivity in multiple sclerosis modelled as connectome stability: A 5-year follow-up study Click here for additional data file. Supplemental material, sj-docx-2-msj-10.1177_13524585211030212 for Functional connectivity in multiple sclerosis modelled as connectome stability: A 5-year follow-up study by Einar August Høgestøl, Samuele Ghezzo, Gro Owren Nygaard, Thomas Espeseth, Piotr Sowa, Mona K Beyer, Hanne Flinstad Harbo, Lars T Westlye, Hanneke E Hulst and Dag Alnæs in Multiple Sclerosis Journal
