# Supplementary material for: Functional connectivity in multiple sclerosis modelled as connectome stability: A 5-year follow-up study
Source: Mult Scler. 2021 Jul 14;28(4):532–40. doi: 10.1177/13524585211030212 (PMC8961247; doi:10.1177/13524585211030212)
Supplement: sj-docx-2-msj-10.1177_13524585211030212 – Supplemental material for Functional connectivity in multiple sclerosis modelled as connectome stability: A 5-year follow-up study [file sj-docx-2-msj-10.1177_13524585211030212.docx]

**SUPPLEMENTAL DATA**

**Disease modifying therapies**

First line treatments included interferons, glatiramer acetate, teriflunomide and dimethyl fumarate, while second line treatments included fingolimod, natalizumab and alemtuzumab.

**Inclusion criteria**

Inclusion criteria included age between 18 and 50 years, ≥ 6 weeks since the last relapse or corticoid treatment at both time points, fluency in Norwegian language, right handedness, no prior neurological or psychiatric disease, no head injury and no substance abuse. We excluded pregnant or breastfeeding women at follow-up and a patient with a previous adverse reaction to gadolinium injection at baseline.

**Fatigue questionnaire**

All subjects completed a fatigue self-report questionnaire (FSS)(1), where FSS mean scores of ≥ 5 were defined as clinically significant fatigue(2).

**Cognitive test battery**

Processing speed was assessed using the Symbol Digit Modalities Test (SDMT) and the time to completion of the first two conditions of the Delis-Kaplan Executive Functioning System Colour Word Interference Test (D-KEFS CWIT)(3-5). In SDMT participants had 90 seconds to pair numbers with specific figures using a reference key, the number of correct responses constituted the raw score of this test. In the first condition of the CWIT participants had to name out loud the colours presented, while in the second condition they had to read the name of colours, raw scores were the seconds needed to complete the tasks. We measured the time to completion of the last two conditions of the D-KEFS CWIT to study executive functioning. We administered the Brief Visuospatial Memory Test-Revised (BVMT-R) to assess visuospatial memory. In this test the participant viewed the stimulus page, consisting of six geometric figures presented in a 2x3 array for ten seconds and was then asked to draw those figures in their correct location on a blank page. The complete battery consisted of six stimulus forms and the number of correct answers determine the total score. Finally, to study verbal memory we used the California Verbal Learning Test-Second Version (CVLT-II)(6). We presented a list of 16 words (List A) five times. After each presentation the participant had to repeat as many words as possible. The sum of all corrected recalls across the five repetitions constitutes the total score. After the fifth trial we presented a new list of 16 words (List B), and participants had to recall items from List A right after presentation of List B (short delay recall). To rule out confounding effects of potential extreme values on our results, performance in specific tests in which patients scored >|3| SD from the mean at baseline were removed from the analyses.

We accessed cross-sectional California Verbal Learning Test-II (CVLT-II) and Colour-Word Interference Test (CWIT) data from the NCNG sample. Due to lack of some cognitive tests in the NCNG sample, we obtained summary statistics for the remaining tests from an Amsterdam HC sample(7). We considered an annual decline of 0.25 SD or above in average cognition and physical performance as significant(7).

**MRI acquisition and structural MRI pre- and post-processing**

Structural MRI data were collected using a 3D T1-weighted Magnetization Prepared Rapid Gradient Echo (MP-RAGE) sequence (TR=2400 ms, TE=3.61 ms, time to inversion=1000 ms, FA=8°, matrix 192x192, field of view=240, scan time 7 minutes and 42 seconds, sagittal slices=160, voxel size =1.20×1.25×1.25 mm). FLAIR sequence parameters were TR=6000 ms, TE=3.33 ms, time to inversion=2200 ms, FOV=260, FA=variable T2, matrix 256 × 204. Each scan lasted 7 minutes and 2 seconds, sagital slices=176 slices, slice thickness=1 mm, voxel size =1.0x1.0x1.0 mm.

For rs-fMRI we used a T2* weighted echo-planar imaging (EPI) sequence (repetition time (TR)=3000 milliseconds (ms), echo time (TE)=70 ms, flip angle (FA)=90°, voxel size=3.44x3.44x4 mm, field-of-view (FOV)=220, descending acquisition, GeneRalized Auto calibrating Partial Acquisition (GRAPPA) acceleration factor=2), 28 transversally oriented slices, no gap, with a scan time of 7 minutes and 30 seconds. The same rs-fMRI acquisition protocol has been used for all MS patients and HC. For fMRI pre-processing and analyses we restricted the rs-fMRI data to the first 100 volumes for all participants to minimize confounding effects due to variability in the number of volumes acquired. Three dummy volumes were collected to avoid T1 saturation effects.

We performed cortical reconstruction by using the T1-weighted scans and volumetric segmentation with FreeSurfer 5.3 (<http://surfer.nmr.mgh.harvard.edu/>)(8). To extract reliable volume and thickness estimates, we used lesion-filled masks at baseline. Lesion-filling was done using the acquired quality controlled lesions masks and the lesions-filling tool in FSL(9). To further validate the brain volume measure, we studied its association with the MS MRI dataset processed with the longitudinal stream in FreeSurfer (10) (Supplemental Table 1 and 2). Lesion volume was based on automatically segmented lesions by Cascade, validated and edited by an experienced neuroradiologist to ensure the highest quality(11). To further validate the lesion output by Cascade we additionally assessed its association with LesionQuant output(12, 13) (Supplementary Table 6).

**fMRI pre-processing**

fMRI analyses were performed using FMRI Expert Analysis Tool (FEAT) Version 6.00, from FMRIB’s Software Library (<https://fsl.fmrib.ox.ac.uk/>)(14). Head motion was corrected using MCFLIRT(15) before linear trends and low-frequency drifts were removed (high-pass filter of 0.01 Hertz). fMRI images were carefully examined by trained research personnel for image artefacts, such as head motion and missing coverage of the scan window. In total, fMRI scans from ten patients and 13 HCs at baseline and eight patients at follow-up were discarded due to incomplete coverage of the visual and parietal cortex. Scans in which the mean relative motion parameter (defined as the average of the translational motion for each scan relative to the previous scan) exceeded 2.5 SD from the average of all runs (across scans) were discarded (none in our MS sample). Brain extraction tool(16) was used to remove non-brain tissue. Spatial smoothing was performed using a Gaussian kernel filter with a full width at half maximum (FWHM) of 6 mm(17). FMRIB’s Nonlinear Image Registration tool (FNIRT) was used to register the participants fMRI volumes to Montreal Neurological Institute (MNI) 152 standard using the T1-weighted scan as an intermediate, which had the non-brain tissue removed using procedures for automated volumetric segmentation in FreeSurfer 5.3 (<http://surfer.nmr.mgh.harvard.edu/>)(8).

Single-session independent component analysis (ICA) was performed for all runs using Multivariate Exploratory Linear Optimized Decomposition into Independent Components (MELODIC)(18). We used FIX(19) for automatic classification of the single-session ICA into signal and noise components, in order to regress noise components from fMRI data. FIX was not used to remove volumes, only as a method to remove noise components in the fMRI data. Data cleaning also included correction based on the estimated motion parameters for each run, using linear regression. These ICA-based procedures for denoising of fMRI-data efficiently reduce motion induced variability, outperforming methods based on regression of motion parameters, scrubbing or de-spiking(20).


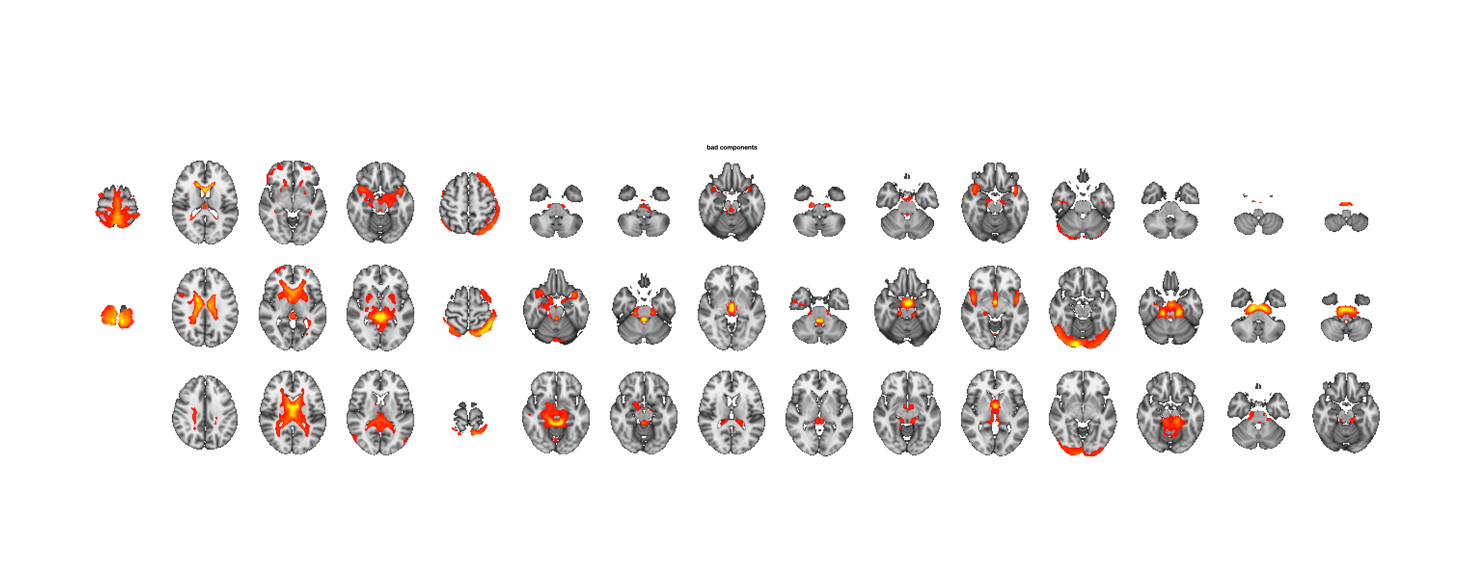


**Supplementary Figure 1** Overview of the 15 bad ICA components. Numbered from left to right; 19, 24, 26, 29, 30, 31, 32, 33, 34, 35, 36, 37, 38, 39, 40.


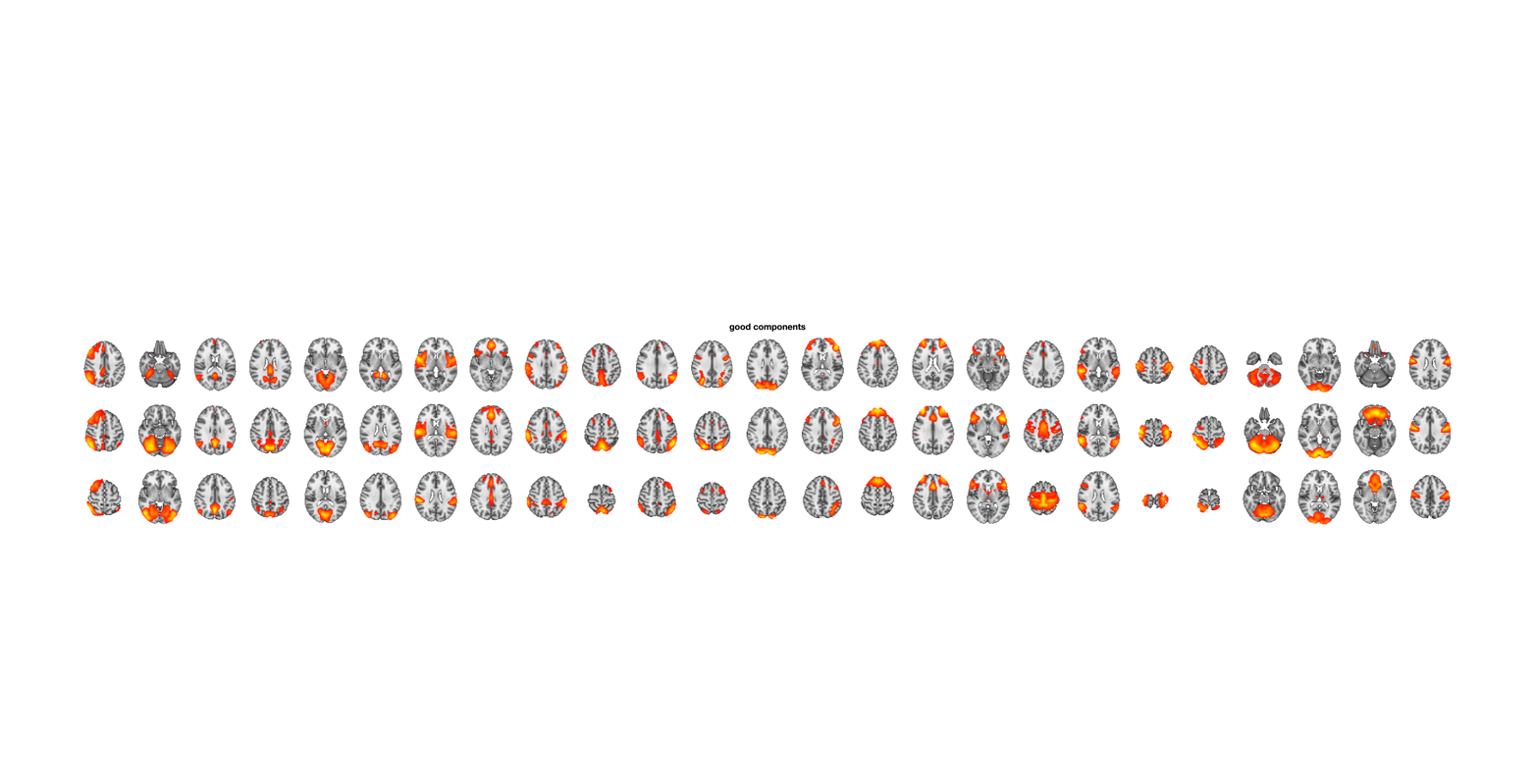


**Supplementary Figure 2** Overview of the 25 good ICA components. Numbered from left to right;1, 2, 3, 4, 5, 6, 7, 8, 9 ,10, 11, 12, 13, 14, 15, 16, 17, 18, 20, 21, 22, 23, 25, 27, 28.

**
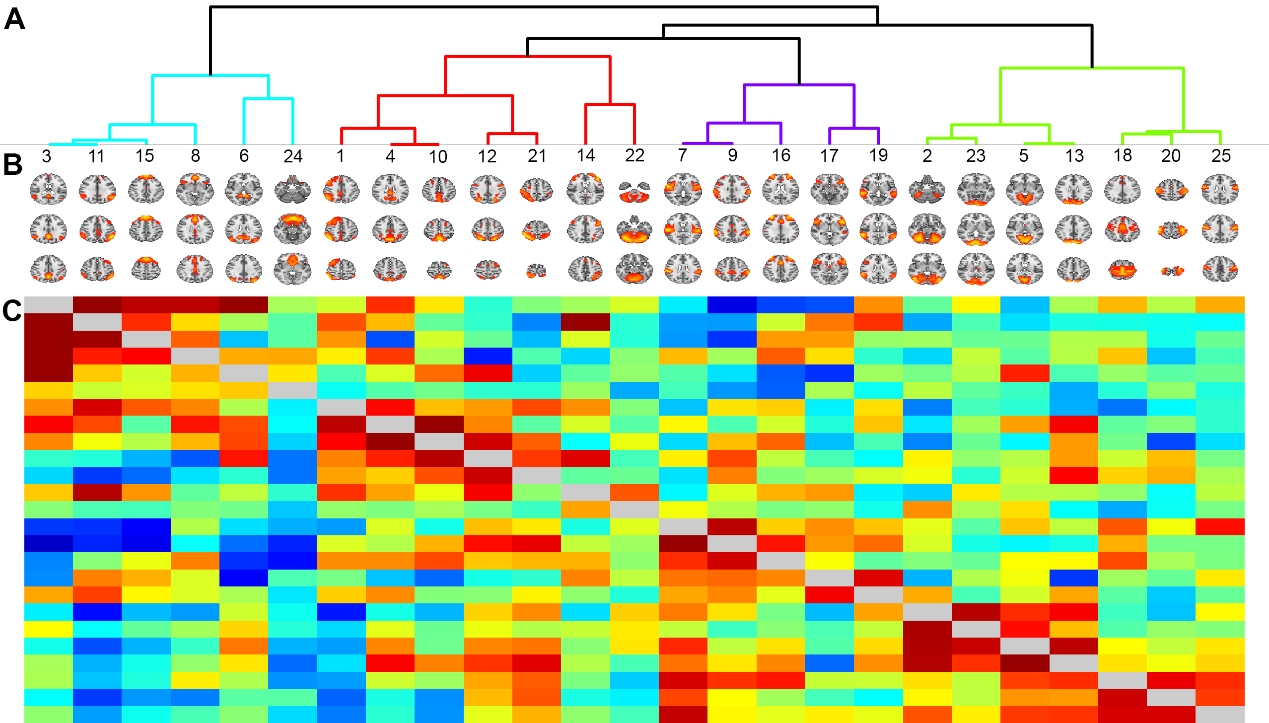
**

**Supplementary Figure 3** Hierarchical clustering of brain nodes. A) Dendrogram showing the clustering of the nodes, based on the full correlations across conditions and participants. (B) The 25 Independent components from group ICA used in the analyses. (C) The full correlation matrix across conditions and participants is shown below the diagonal. Regularized partial correlation across conditions are presented above the diagonal. Warm colours indicate positive correlations, cold colours negative correlations.

**Supplementary Table 1** Anatomical description of independent components (Harvard-Oxford Cortical Structural Atlas).

| **Networks** | **Anatomical definition** |
| --- | --- |
| ***(1) Nodes clustering with DMN and frontoparietal networks*** | |
| IC 3 | Cingulate gyrus posterior division, precuneus cortex, lateral occipital cortex |
| IC 6 | Lateral occipital cortex, precuneus cortex |
| IC 8 | Cingulate gyrus, paracingulate gyrus, frontal orbital cortex, putamen, caudate nucleus |
| IC 11 | Lateral occipital cortex, angular gyrus, cingulate gyrus |
| IC 15 | Frontal pole, superior frontal gyrus |
| IC 24 | Frontal medial cortex, frontal orbital cortex, subcallosal cortex |
| ***(2) Nodes clustering DMN and frontoparietal networks*** | |
| IC 1 | Angular gyrus, frontal pole, precuneus, cingulate gyrus, middle temporal gyrus |
| IC 4 | Precuneus cortex, cingulate gyrus, angular gyrus |
| IC 10 | Occipital cortex, precuneus cortex, superior frontal gyrus |
| IC 12 | Lateral occipital cortex superior division, superior parietal lobule, supramarginal gyrus posterior division, precentral gyrus |
| IC 14 | Frontal pole, supramarginal gyrus posterior division |
| IC 21 | Lateral occipital cortex, superior parietal lobule |
| IC 22 | Cerebellum, thalamus, putamen, caudate nucleus |
| ***(3) Nodes clustering with Auditory networks*** | |
| IC 7 | Opercular cortex |
| IC 9 | Supramarginal gyrus, precentral gyrus, cingulate gyrus |
| IC 16 | Frontal pole, cingulate gyrus anterior division |
| IC 17 | Inferior frontal gyrus, caudate nucleus, hippocampus. amygdala |
| IC 19 | Temporal gyrus |
| ***(4) Nodes clustering with sensory/motor networks*** | |
| IC 2 | Occipital fusiform gyrus, parahippocampal gyrus. Hippocampus, amygdala |
| IC 5 | Lingual gyrus, intra-calcarine, cuneal cortex, |
| IC 13 | Lateral occipital cortex, cuneal cortex |
| IC 18 | Precuneus cortex, precentral gyrus, postcentral gyrus, thalamus, caudate nucleus |
| IC 20 | Postcentral gyrus |
| IC 23 | Occipital pole, thalamus |
| IC 25 | Precentral gyrus, postcentral gyrus |

**Supplementary Table 2** Demographic and clinical characteristics based on NEDA status at follow-up

|  | **No evidence of disease activity** | **Evidence of disease activity** |
| --- | --- | --- |
| **(a) Demographic characteristics** | n=27 | n= 35 |
| Female, n (%) | 18 (67) | 26 (74) |
| Age, mean years (SD) | 39.5 (7.0) | 39.1 (7.3) |
| Disease duration, mean months (SD) | 142 (69) | 112 (50) |
| Age at first symptom, mean years (SD) | 30.4.3 (6.8) | 27.1 (6.7) |
| ***Disease modifying treatment*** |  |  |
| None, n (%) | 11 (41) | 8 (23) |
| First line treatment, n (%) | 9 (33) | 14 (40) |
| Second line treatment, n (%) | 7 (26) | 13 (37) |
| **(b) Clinical evaluation** |  |  |
| ***Multiple sclerosis classification*** |  |  |
| RRMS, n (%) | 26 (96) | 34 (97) |
| PPMS, n (%) | 1 (4) |  |
| SPMS, n (%) |  | 1 (3) |
| ***Neurological disability*** |  |  |
| EDSS, median (SD, range) | 1.50 (1.0, 0-4) | 2.5 (1.3, 0-6) |
| MSSS (SD) | 1.7 (1.2) | 3.2 (1.8) |
| Relapses, median (range) |  | 1.0 (0-3) |
| FSS, mean (SD) | 4.0 (1.9) | 4.3 (1.9) |

**Supplementary Table 3** Clinical evaluation and MRI data of the MS sample.

|  | **Baseline** | **Follow-up** | **P value** |
| --- | --- | --- | --- |
| **(a) Cognitive assessment** |  |  |  |
| *Processing speed* |  |  |  |
| SDMT, mean correct (SD) | 52.4 (9.6) | 54.5 (10.9) | 0.13 |
| CWIT colour naming, mean seconds (SD) | 29.7 (5.2) | 27.42 (4.1) | <0.001 |
| CWIT reading colour names, mean seconds (SD) | 22.8 (4.6) | 21.04 (3.5) | <0.001 |
| ***Verbal memory*** |  |  |  |
| CVLT list A, five repetitions, mean correct (SD) | 61.6 (10.8) | 64.9 (10.5) | 0.02 |
| CVLT list A short delay recall, mean correct (SD) | 13.7 (2.6) | 14.0 ( 2.5) | 0.96 |
| ***Visuospatial memory*** |  |  |  |
| BVMT-R, mean correct (SD) | 29.2 (5.7) | 29.7 (4.8) | 0.18 |
| ***Executive functioning*** |  |  |  |
| CWIT colour-word inhibition, mean seconds (SD) | 49.8 (13.4) | 45.8 (9.5) | 0.03 |
| CWIT colour-word inhibition switching, mean seconds (SD) | 59.4 (18.9) | 55.4 (11.1) | 0.03 |
| **(b) Physical assessment** |  |  |  |
| T25-FW, mean seconds (SD) | 4.0 (0.7) | 4.0 (1.1) | 0.89 |
| 9-hole peg test dominant hand, mean seconds (SD) | 200.0 (3.1) | 20.6 (8.4) | 0.58 |
| **(c) Brain volume** |  |  |  |
| Lesion filled volume, mean ml (SD) | 1137.2 (95.6) |  |  |
| Raw volume, mean ml (SD) | 1137.9 (95.1) | 1118.9 (98.3) |  |

**
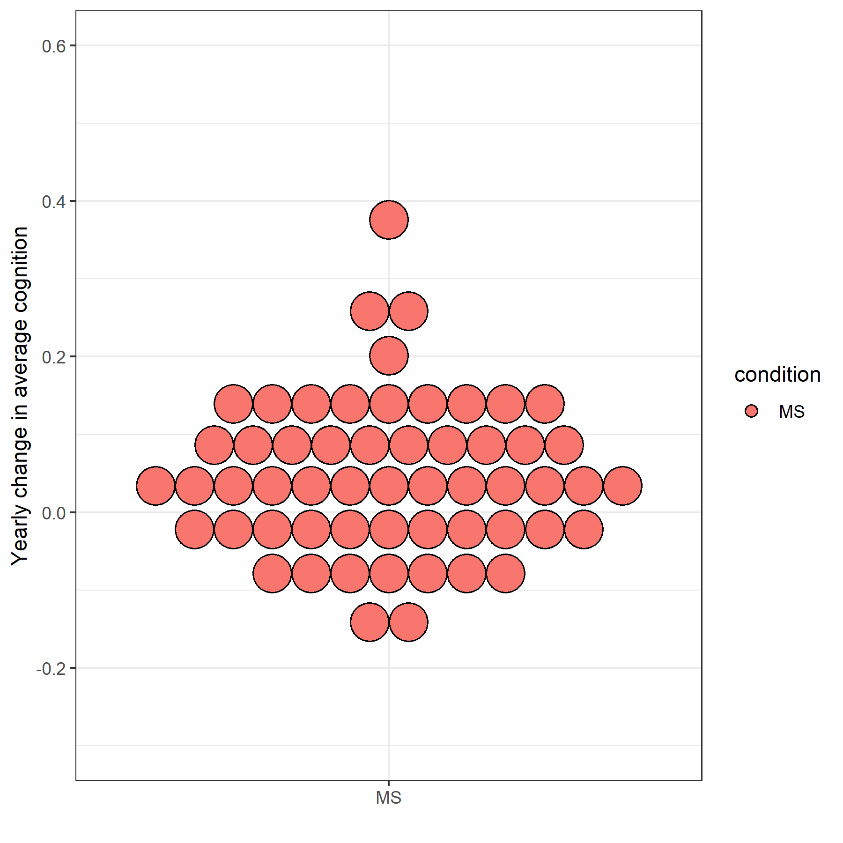
**

**Supplementary Figure 4** Average annual change in cognition in the MS cohort at follow-up. In the Y-axis is represented the change in SD between baseline and follow-up for the measure of overall cognition in the MS cohort. Significant change >0.25.


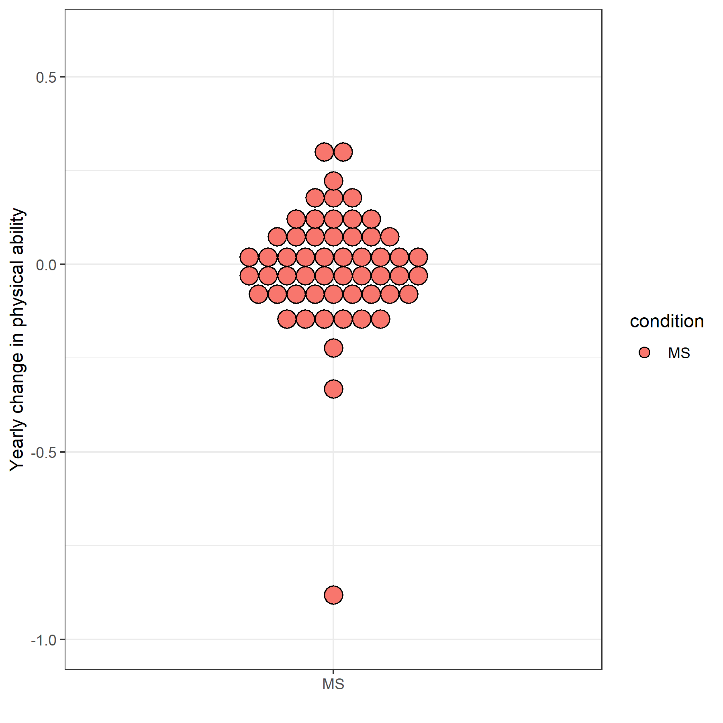


**Supplementary Figure 5** Average annual change in physical ability in the MS cohort at follow-up. In the Y-axis is represented the change in SD between baseline and follow-up for the measure of overall physical ability in the MS cohort. Significant change >0.25.

**Supplementary Table 4** Correlation of motion parameters with clinical variables.

| **Association** | **Pearson ρ** | **P value** |
| --- | --- | --- |
| Average cognition at follow-up: Signal to noise ratio | -0.12 | .45 |
| Average cognition at follow-up: Mean relative motion | 0.14 | .40 |
| Change in cognition: Signal to noise ratio | 0.02 | .88 |
| Change in cognition: Mean relative motion | -0.02 | .90 |
| Physical performance at follow-up: Signal to noise ratio | -0.15 | .36 |
| Physical performance at follow-up: Mean relative motion | -0.01 | .93 |
| Change in physical performance: Signal to noise ratio | -0.05 | .75 |
| Change in physical performance: Mean relative motion | -0.08 | .61 |

|  | **1** | **2** | **3** | **4** | **5** | **6** | **7** | **8** | **9** | **10** | **11** | **12** | **13** | **14** | **15** | **16** | **17** | **18** | **19** | **20** | **21** | **22** | **23** | **24** | **25** |
| --- | --- | --- | --- | --- | --- | --- | --- | --- | --- | --- | --- | --- | --- | --- | --- | --- | --- | --- | --- | --- | --- | --- | --- | --- | --- |
| **1** |  |  |  |  |  |  |  |  |  |  |  |  |  |  |  |  |  |  |  |  |  |  |  |  |  |
| **2** | 0.702 |  |  |  |  |  |  |  |  |  |  |  |  |  |  |  |  |  |  |  |  |  |  |  |  |
| **3** | 0.065 | 0.25 |  |  |  |  |  |  |  |  |  |  |  |  |  |  |  |  |  |  |  |  |  |  |  |
| **4** | 0.823 | 0.849 | 0.982 |  |  |  |  |  |  |  |  |  |  |  |  |  |  |  |  |  |  |  |  |  |  |
| **5** | 0.328 | 0.699 | 0.605 | 0.783 |  |  |  |  |  |  |  |  |  |  |  |  |  |  |  |  |  |  |  |  |  |
| **6** | 0.211 | 0.001 | 0.239 | 0.139 | 0.996 |  |  |  |  |  |  |  |  |  |  |  |  |  |  |  |  |  |  |  |  |
| **7** | 0.813 | 0 | 0.795 | 0.537 | 0.211 | 0.373 |  |  |  |  |  |  |  |  |  |  |  |  |  |  |  |  |  |  |  |
| **8** | 0.118 | 0.156 | 0.843 | 0.174 | 0.758 | 0.025 | 0.718 |  |  |  |  |  |  |  |  |  |  |  |  |  |  |  |  |  |  |
| **9** | 0.976 | 0.003 | 0.482 | 0.188 | 0.904 | 0.342 | 0.835 | 0.239 |  |  |  |  |  |  |  |  |  |  |  |  |  |  |  |  |  |
| **10** | 0.146 | 0.131 | 0.006 | 0.025 | 0.345 | 0.393 | 0.473 | 0.086 | 0.601 |  |  |  |  |  |  |  |  |  |  |  |  |  |  |  |  |
| **11** | 0.058 | 0.368 | 0.758 | 0.018 | 0.95 | 0.001 | 0.01 | 0.275 | 0.75 | 0.371 |  |  |  |  |  |  |  |  |  |  |  |  |  |  |  |
| **12** | 0.864 | 0.71 | 0.963 | 0.364 | 0.879 | 0.122 | 0.109 | 0.047 | 0.695 | 0.04 | 0.021 |  |  |  |  |  |  |  |  |  |  |  |  |  |  |
| **13** | 0.913 | 0.727 | 0.06 | 0.456 | 0.133 | 0.431 | 0.689 | 0.348 | 0.308 | 0.166 | 0.872 | 0.24 |  |  |  |  |  |  |  |  |  |  |  |  |  |
| **14** | 0.089 | 0.195 | 0.127 | 0.139 | 0.604 | 0.194 | 0.551 | 0.36 | 0.97 | 0 | 0.012 | 0.387 | 0.463 |  |  |  |  |  |  |  |  |  |  |  |  |
| **15** | 0.622 | 0.129 | 0.013 | 0.026 | 0.079 | 0.708 | 0.434 | 0.025 | 0.252 | 0.935 | 0 | 0.717 | 0.791 | 0.197 |  |  |  |  |  |  |  |  |  |  |  |
| **16** | 0.95 | 0.284 | 0.109 | 0.07 | 0.048 | 0.695 | 0 | 0.256 | 0.016 | 0.194 | 0.011 | 0.116 | 0.327 | 0.049 | 0.157 |  |  |  |  |  |  |  |  |  |  |
| **17** | 0.959 | 0.003 | 0.112 | 0.805 | 0.92 | 0.589 | 0.318 | 0.16 | 0.23 | 0.231 | 0.074 | 0.512 | 0.49 | 0.019 | 0.488 | 0.003 |  |  |  |  |  |  |  |  |  |
| **18** | 0.313 | 0.005 | 0.944 | 0.872 | 0.802 | 0.278 | 0.527 | 0.001 | 0.887 | 0.893 | 0.573 | 0.113 | 0.184 | 0.528 | 0.465 | 0.351 | 0.67 |  |  |  |  |  |  |  |  |
| **19** | 0.508 | 0.068 | 0.529 | 0.003 | 0.375 | 0.816 | 0.351 | 0.043 | 0.726 | 0.642 | 0.731 | 0.507 | 0.228 | 0.454 | 0.432 | 0.064 | 0.171 | 0.623 |  |  |  |  |  |  |  |
| **20** | 0.113 | 0.993 | 0.369 | 0.233 | 0.064 | 0.011 | 0.064 | 0.555 | 0.66 | 0.937 | 0.13 | 0.469 | 0.503 | 0.026 | 0.686 | 0.253 | 0.12 | 0.26 | 0.09 |  |  |  |  |  |  |
| **21** | 0.28 | 0.475 | 0.705 | 0.978 | 0.546 | 0.446 | 0.347 | 0.223 | 0.096 | 0.851 | 0.047 | 0.83 | 0.537 | 0.209 | 0.71 | 0.613 | 0.824 | 0.322 | 0.174 | 0.977 |  |  |  |  |  |
| **22** | 0.32 | 0.588 | 0.329 | 0.368 | 0.114 | 0.95 | 0.004 | 0.892 | 0.839 | 0.157 | 0.013 | 0.037 | 0.528 | 0.003 | 0.028 | 0.15 | 0.918 | 0.73 | 0.427 | 0.876 | 0.464 |  |  |  |  |
| **23** | 0.588 | 0.307 | 0.857 | 0.058 | 0.975 | 0.333 | 0.182 | 0.175 | 0.467 | 0.32 | 0.103 | 0.075 | 0.226 | 0.075 | 0.824 | 0.613 | 0.147 | 0.286 | 0.278 | 0.019 | 0.838 | 0.235 |  |  |  |
| **24** | 0.265 | 0.265 | 0.51 | 0.952 | 0.98 | 0.759 | 0.144 | 0.014 | 0.118 | 0.132 | 0.486 | 0.591 | 0.746 | 0.485 | 0.045 | 0.921 | 0.118 | 0.069 | 0.51 | 0.212 | 0.999 | 0.958 | 0.423 |  |  |
| **25** | 0.328 | 0.599 | 0.766 | 0.308 | 0.554 | 0.228 | 0.63 | 0.898 | 0.92 | 0.909 | 0.317 | 0.007 | 0.74 | 0.532 | 0.298 | 0.564 | 0.832 | 0.419 | 0.034 | 0.867 | 0.099 | 0.741 | 0.579 | 0.455 |  |

**Supplementary Figure 6** Edgewise analysis of functional connectivity (FC) abnormalities in MS vs HCs. Uncorrected p-values form multivariate linear regressions assessing differences in FC at the level of single connections between MS and HCs.


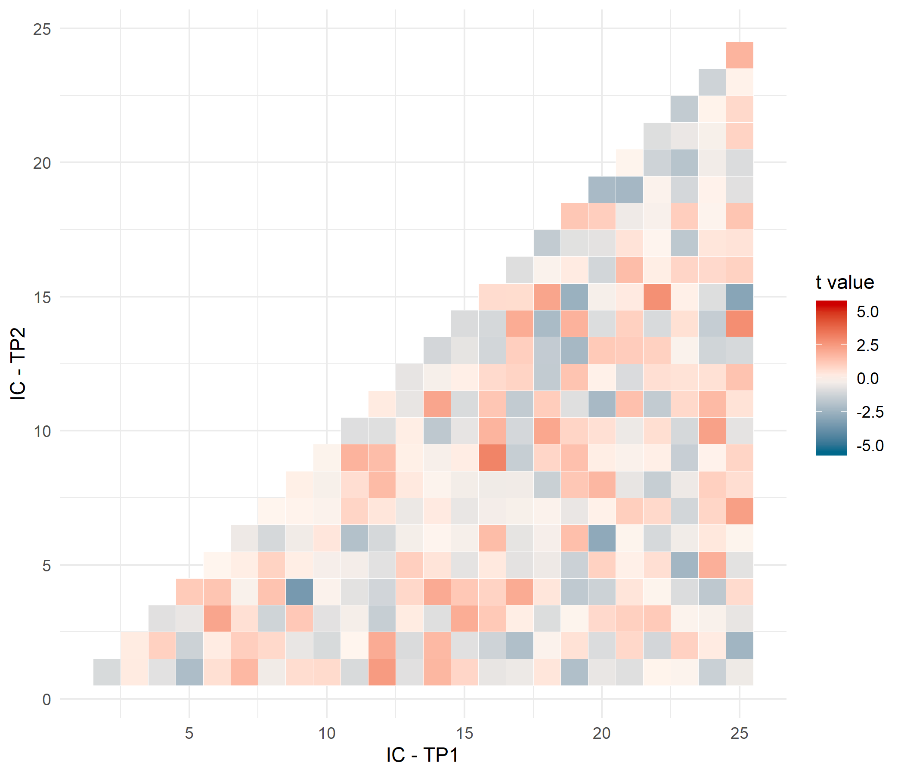


**Supplementary Figure 7** Functional connectivity (FC) changes over time in MS patients at the level of single edges. Each square represents a t-test between ICs at the two time points. Red colours indicate an increase in FC at follow-up, while blue colours a represents a decrease.

|  | **1** | **2** | **3** | **4** | **5** | **6** | **7** | **8** | **9** | **10** | **11** | **12** | **13** | **14** | **15** | **16** | **17** | **18** | **19** | **20** | **21** | **22** | **23** | **24** | **25** |
| --- | --- | --- | --- | --- | --- | --- | --- | --- | --- | --- | --- | --- | --- | --- | --- | --- | --- | --- | --- | --- | --- | --- | --- | --- | --- |
| **1** |  |  |  |  |  |  |  |  |  |  |  |  |  |  |  |  |  |  |  |  |  |  |  |  |  |
| **2** | 0.314 |  |  |  |  |  |  |  |  |  |  |  |  |  |  |  |  |  |  |  |  |  |  |  |  |
| **3** | 0.777 | 0.799 |  |  |  |  |  |  |  |  |  |  |  |  |  |  |  |  |  |  |  |  |  |  |  |
| **4** | 0.469 | 0.324 | 0.446 |  |  |  |  |  |  |  |  |  |  |  |  |  |  |  |  |  |  |  |  |  |  |
| **5** | 0.039 | 0.194 | 0.59 | 0.248 |  |  |  |  |  |  |  |  |  |  |  |  |  |  |  |  |  |  |  |  |  |
| **6** | 0.559 | 0.815 | 0.031 | 0.188 | 0.997 |  |  |  |  |  |  |  |  |  |  |  |  |  |  |  |  |  |  |  |  |
| **7** | 0.1 | 0.293 | 0.565 | 0.871 | 0.851 | 0.658 |  |  |  |  |  |  |  |  |  |  |  |  |  |  |  |  |  |  |  |
| **8** | 0.726 | 0.448 | 0.223 | 0.174 | 0.352 | 0.285 | 1 |  |  |  |  |  |  |  |  |  |  |  |  |  |  |  |  |  |  |
| **9** | 0.515 | 0.573 | 0.223 | 0.001 | 0.845 | 0.719 | 0.967 | 0.882 |  |  |  |  |  |  |  |  |  |  |  |  |  |  |  |  |  |
| **10** | 0.475 | 0.316 | 0.473 | 0.91 | 0.812 | 0.689 | 0.911 | 0.842 | 0.931 |  |  |  |  |  |  |  |  |  |  |  |  |  |  |  |  |
| **11** | 0.318 | 0.999 | 0.799 | 0.486 | 0.779 | 0.055 | 0.384 | 0.539 | 0.073 | 0.41 |  |  |  |  |  |  |  |  |  |  |  |  |  |  |  |
| **12** | 0.016 | 0.045 | 0.148 | 0.247 | 0.469 | 0.282 | 0.681 | 0.114 | 0.128 | 0.413 | 0.776 |  |  |  |  |  |  |  |  |  |  |  |  |  |  |
| **13** | 0.443 | 0.344 | 0.796 | 0.433 | 0.299 | 0.787 | 0.638 | 0.744 | 0.886 | 0.844 | 0.562 | 0.546 |  |  |  |  |  |  |  |  |  |  |  |  |  |
| **14** | 0.09 | 0.104 | 0.425 | 0.043 | 0.644 | 0.981 | 0.772 | 0.94 | 0.831 | 0.098 | 0.031 | 0.818 | 0.26 |  |  |  |  |  |  |  |  |  |  |  |  |
| **15** | 0.4 | 0.44 | 0.05 | 0.215 | 0.511 | 0.825 | 0.577 | 0.765 | 0.825 | 0.54 | 0.339 | 0.875 | 0.533 | 0.343 |  |  |  |  |  |  |  |  |  |  |  |
| **16** | 0.563 | 0.211 | 0.226 | 0.355 | 0.737 | 0.125 | 0.782 | 0.706 | 0.003 | 0.076 | 0.198 | 0.469 | 0.255 | 0.291 | 0.501 |  |  |  |  |  |  |  |  |  |  |
| **17** | 0.657 | 0.045 | 0.849 | 0.044 | 0.494 | 0.522 | 0.846 | 0.716 | 0.152 | 0.276 | 0.123 | 0.369 | 0.299 | 0.047 | 0.505 | 0.4 |  |  |  |  |  |  |  |  |  |
| **18** | 0.674 | 0.927 | 0.373 | 0.674 | 0.673 | 0.81 | 0.912 | 0.169 | 0.404 | 0.034 | 0.274 | 0.121 | 0.127 | 0.032 | 0.03 | 0.907 | 0.121 |  |  |  |  |  |  |  |  |
| **19** | 0.046 | 0.605 | 0.961 | 0.104 | 0.187 | 0.14 | 0.593 | 0.213 | 0.15 | 0.386 | 0.419 | 0.182 | 0.024 | 0.07 | 0.012 | 0.787 | 0.454 | 0.21 |  |  |  |  |  |  |  |
| **20** | 0.579 | 0.363 | 0.453 | 0.197 | 0.337 | 0.005 | 0.92 | 0.092 | 0.849 | 0.573 | 0.028 | 0.908 | 0.238 | 0.379 | 0.817 | 0.258 | 0.486 | 0.293 | 0.03 |  |  |  |  |  |  |
| **21** | 0.418 | 0.432 | 0.316 | 0.661 | 0.898 | 0.964 | 0.295 | 0.56 | 0.891 | 0.657 | 0.152 | 0.348 | 0.269 | 0.322 | 0.774 | 0.127 | 0.626 | 0.689 | 0.023 | 0.974 |  |  |  |  |  |
| **22** | 0.968 | 0.262 | 0.24 | 0.958 | 0.55 | 0.296 | 0.439 | 0.152 | 0.877 | 0.541 | 0.123 | 0.554 | 0.325 | 0.323 | 0.007 | 0.85 | 0.982 | 0.858 | 0.914 | 0.211 | 0.392 |  |  |  |  |
| **23** | 0.941 | 0.332 | 0.94 | 0.367 | 0.02 | 0.731 | 0.258 | 0.663 | 0.157 | 0.282 | 0.447 | 0.622 | 0.899 | 0.577 | 0.891 | 0.39 | 0.094 | 0.288 | 0.275 | 0.068 | 0.61 | 0.12 |  |  |  |
| **24** | 0.172 | 0.787 | 0.879 | 0.098 | 0.059 | 0.719 | 0.394 | 0.309 | 0.943 | 0.024 | 0.106 | 0.581 | 0.237 | 0.139 | 0.394 | 0.453 | 0.679 | 0.954 | 0.941 | 0.736 | 0.843 | 0.956 | 0.215 |  |  |
| **25** | 0.676 | 0.018 | 0.562 | 0.483 | 0.482 | 0.96 | 0.02 | 0.537 | 0.382 | 0.511 | 0.622 | 0.173 | 0.304 | 0.006 | 0.003 | 0.334 | 0.627 | 0.189 | 0.441 | 0.366 | 0.36 | 0.441 | 0.936 | 0.078 |  |

**Supplementary Figure 8** Functional connectivity changes over time in MS patients at the level of single edges. Uncorrected p value table of functional connectivity changes over time in MS patients at the level of single edges measured by paired sample t-tests.

**Supplementary Table 5** Within network functional connectivity changes over time in MS patients.

|  | **Beta coefficient** | **T value** | **Standard deviation** | **P value** |
| --- | --- | --- | --- | --- |
| **Full brain** | -0.0002 | -0.18 | 0.001 | .86 |
| **Network 1** DMN and frontoparietal nodes | 0.001 | 0.24 | 0.006 | .81 |
| **Network 2** DMN and frontoparietal nodes | 0.0009 | 0.24 | 0.004 | .81 |
| **Network 3** Auditory nodes | 0.002 | 0.26 | 0.007 | .81 |
| **Network 4** Sensory and motor nodes | -0.008 | -1.49 | 0.005 | .55 |
| ^Results of multivariate linear regression models corrected for sex, age, mean relative motion and signal to noise ratio. P values corrected for multiple testing by false discovery rate. * P value significant after correction for multiple testing by false discovery rate^ | | | | |

**Supplementary Figure 9** Overview of the resulting correlations between lesion filled data (after careful lesion masking during the estimation of the node time series at baseline (n=60)) with data not accounting for the lesions. (A) Pearson correlation coefficient based on full correlations. (B) Spearman correlation coefficient based on full correlations. (C) Heatmap illustrating the t-values of the differences between estimates based on full correlations. (D) Pearson correlation coefficient based on partial correlations. (E) Spearman correlation coefficient based on partial correlations. (F) Heatmap illustrating the t-values of the difference between estimates based on partial correlations.

**Supplementary Table 6** Connectome stability EDA vs NEDA patients.

|  | **Beta coefficient** | **T-value** | **SD** | **P-value (raw)** | **P-value (corrected)** |
| --- | --- | --- | --- | --- | --- |
| **Full brain** | 0.013 | 0.66 | 0.02 | .51 | 1 |
| **Network 1** DMN and frontoparietal nodes | 0.05 | 0.70 | 0.07 | .49 | .94 |
| **Network 2** DMN and frontoparietal nodes | 0.14 | 2.26 | 0.03 | .03 | .22 |
| **Network 3** Auditory nodes | 0.07 | 0.84 | 0.09 | .41 | .78 |
| **Network 4** Sensory and motor nodes | -0.006 | -0.09 | 0.06 | .92 | 1 |


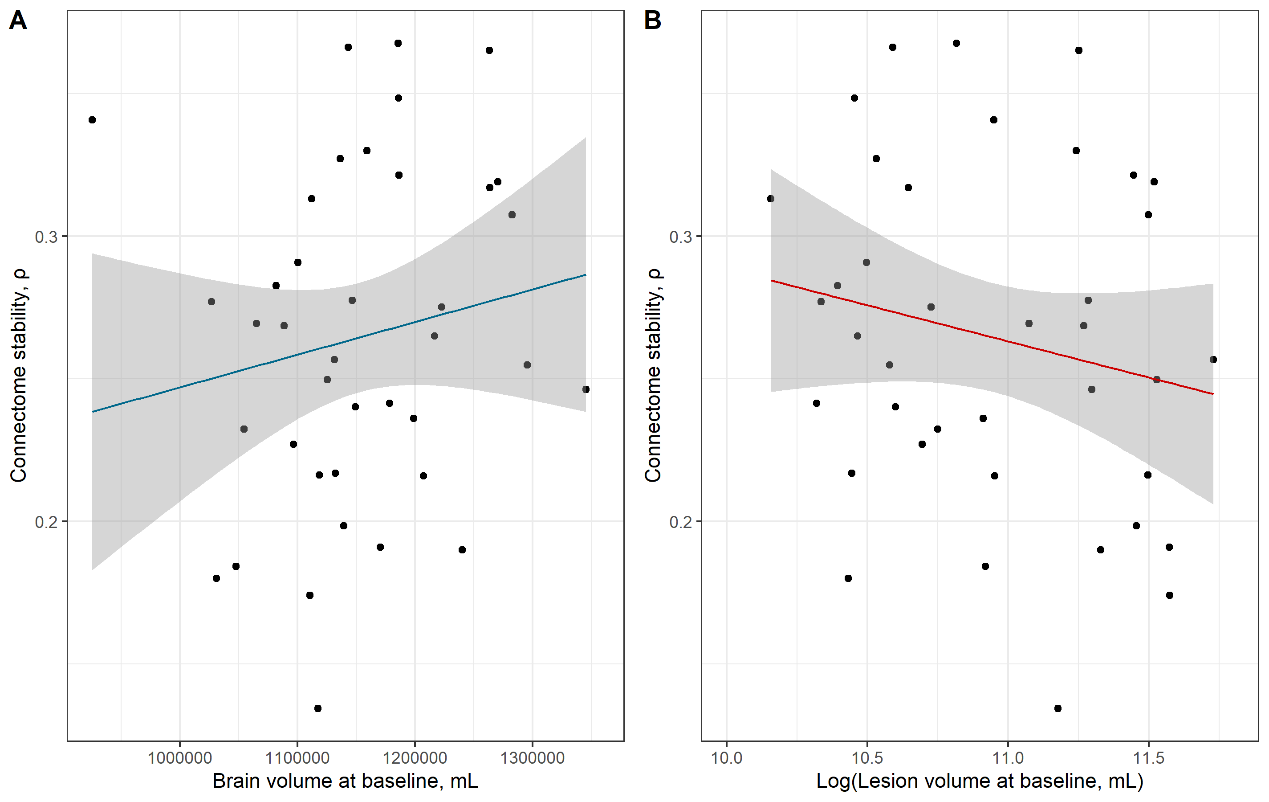


**Supplementary Figure 10** Correlation between structural damage and stability of the brain functional connectome. (A) Correlation between brain volume at baseline and the rate of FC reorganization, ρ=0.16, p=0.32. (B) Correlation between lesion load at baseline and the rate of FC reorganization, ρ=-0.19, p=0.24**.**

**

**

**Supplementary Figure 11** Correlation between changes in structural damage and stability of the brain functional connectome. (A) Correlation between brain atrophy and the rate of FC reorganization, ρ =0,40, p=0,06. (B) Correlation between lesion volume change at baseline and the rate of FC reorganization, ρ =0,13, p=0,63.

**Supplementary Table 7** Correlation between different estimates of brain and lesion volumes.

| **Association** | **Pearson ρ** | **P-value** |
| --- | --- | --- |
| Lesion filled brain volume: brain volume raw | 0.99 | 2.2e-16 |
| Lesion filled brain volume: longitudinal brain volume t1 | 0.99 | 2.2e-16 |
| Brain volume raw: longitudinal brain volume t1 | 0.99 | 2.2e-16 |
| Cascade lesion volume: LesionQuant lesion volume | 0.79 | 3.04e-14 |

**Supplementary Table 8** Overview of the comparisons of the signal components from the 40 and 50 IC decompositions.

| **The 25 signal components in d40 decomposition, their spatial correlation with d50 decomposition, and d40/d50 correlations with Smith et al. Networks** | | | | | | | | | | |
| --- | --- | --- | --- | --- | --- | --- | --- | --- | --- | --- |
| **d40-ICs** | **d-40_Smith-r** | **d-40_Smith-IC** | **d50-d40-r** | **d50-d40-IC** | **d50-Smith-r** | **d50-Smith-IC** | **d40-SmithNetwork** | **d50-SmithNetwork** | **d40/d50 Corresponds** |  |
| **1** | 0,58105764 | 9 | 0,805599076 | 5 | 0,464650174 | 9 | Frontoparietal - Smith et al. network map 9(20) | Frontoparietal - Smith et al. network map 9(20) | TRUE |  |
| **2** | 0,691060916 | 3 | 0,738592785 | 3 | 0,475350366 | 3 | Visual-3 Smith et al.network map 3(20) | Visual-3 Smith et al.network map 3(20) | TRUE |  |
| **3** | 0,627597813 | 4 | 0,850545255 | 1 | 0,602868177 | 4 | DMN - Smith et al. network map 4(20) | DMN - Smith et al. network map 4(20) | TRUE |  |
| **4** | 0,379736657 | 4 | 0,913116923 | 14 | 0,390913743 | 4 | DMN - Smith et al. network map 4(20) | DMN - Smith et al. network map 4(20) | TRUE |  |
| **5** | 0,77841377 | 1 | 0,860266615 | 2 | 0,721555565 | 1 | Visual-1 Smith et al. network map 1(20 ) | Visual-1 Smith et al. network map 1(20 ) | TRUE |  |
| **6** | 0,372426963 | 4 | 0,818691442 | 6 | 0,363357184 | 4 | DMN - Smith et al. network map 4(20) | DMN - Smith et al. network map 4(20) | TRUE |  |
| **7** | 0,621509293 | 7 | 0,857387009 | 7 | 0,54286573 | 7 | Auditory – Smith et al. network map 7(20) | Auditory – Smith et al. network map 7(20) | TRUE |  |
| **8** | 0,574883382 | 8 | 0,85799634 | 10 | 0,578703759 | 8 | Executive control – Smith et al. network map 8(20) | Executive control – Smith et al. network map 8(20) | TRUE |  |
| **9** | 0,278153038 | 9 | 0,649854855 | 18 | 0,342340247 | 9 | Frontoparietal - Smith et al. network map 9(20) | Frontoparietal - Smith et al. network map 9(20) | TRUE |  |
| **10** | 0,109447322 | 5 | 0,800835667 | 13 | 0,070787419 | 3 | Cerebellum - Smith et al. network map 5(20) | Visual-3 Smith et al.network map 3(20) | NOT TRUE |  |
| **11** | 0,343101131 | 10 | 0,567481914 | 8 | 0,467119034 | 10 | Frontoparietal – Smith et al. network map 10(20) | Frontoparietal – Smith et al. network map 10(20) | TRUE |  |
| **12** | 0,362252071 | 10 | 0,668505225 | 17 | 0,337148971 | 3 | Frontoparietal – Smith et al. network map 10(20) | Visual-3 Smith et al.network map 3(20) | NOT TRUE |  |
| **13** | 0,158365299 | 1 | 0,791872567 | 9 | 0,209468794 | 1 | Visual-1 Smith et al. network map 1(20 ) | Visual-1 Smith et al. network map 1(20 ) | TRUE |  |
| **14** | 0,521687253 | 10 | 0,508007109 | 25 | 0,346430581 | 10 | Frontoparietal – Smith et al. network map 10(20) | Frontoparietal – Smith et al. network map 10(20) | TRUE |  |
| **15** | 0,084007096 | 3 | 0,740773724 | 11 | 0,136627382 | 9 | Visual-3 Smith et al.network map 3(20) | Frontoparietal - Smith et al. network map 9(20) | NOT TRUE |  |
| **16** | 0,3734035 | 8 | 0,72540456 | 21 | 0,256386236 | 8 | Executive control – Smith et al. network map 8(20) | Executive control – Smith et al. network map 8(20) | TRUE |  |
| **17** | 0,36485525 | 7 | 0,586733541 | 22 | 0,358724688 | 10 | Auditory – Smith et al. network map 7(20) | Frontoparietal – Smith et al. network map 10(20) | NOT TRUE |  |
| **18** | 0,528885005 | 6 | 0,805698276 | 15 | 0,406701364 | 6 | Sensorimotor - Smith et al. network map 6(20) | Sensorimotor - Smith et al. network map 6(20) | TRUE |  |
| **19** | 0,329703755 | 7 | 0,732470128 | 30 | 0,352770176 | 7 | Auditory – Smith et al. network map 7(20) | Auditory – Smith et al. network map 7(20) | TRUE |  |
| **20** | 0,24792826 | 6 | 0,748952061 | 36 | 0,189513983 | 6 | Sensorimotor - Smith et al. network map 6(20) | Sensorimotor - Smith et al. network map 6(20) | TRUE |  |
| **21** | 0,203979232 | 3 | 0,524515499 | 26 | 0,429504221 | 6 | Visual-3 Smith et al.network map 3(20) | Sensorimotor - Smith et al. network map 6(20) | NOT TRUE |  |
| **22** | 0,493164908 | 5 | 0,677422867 | 23 | 0,622602867 | 5 | Cerebellum - Smith et al. network map 5(20) | Cerebellum - Smith et al. network map 5(20) | TRUE |  |
| **23** | 0,674709692 | 2 | 0,820774911 | 31 | 0,620865881 | 2 | Visual-2 Smith et al.network map 2(20) | Visual-2 Smith et al.network map 2(20) | TRUE |  |
| **24** | 0,147540039 | 8 | 0,841810919 | 20 | 0,157074475 | 8 | Executive control – Smith et al. network map 8(20) | Executive control – Smith et al. network map 8(20) | TRUE |  |
| **25** | 0,291658386 | 6 | 0,924146069 | 28 | 0,287638182 | 6 | Sensorimotor - Smith et al. network map 6(20) | Sensorimotor - Smith et al. network map 6(20) | TRUE |  |
|  |  |  |  |  |  |  |  |  |  |  |
| **The 25 d50 components not among by best matches for the 25 signal components in the d40 decomposition** | | | | | | | | | | |
| **d50_IC** | **d_50_Smith_r** | **d_50_Smith_IC** | **d40_d50_r** | **d40_d50_IC** | **d40_Smith_r** | **d40_Smith_IC** | **d50_SmithNetwork** | **d40_SmithNetwork** | **d50/d40 Corresponds** | **d40-match is a noise-IC** |
| **12** | 0,069380959 | 5 | 0,479701874 | 23 | 0,493164908 | 5 | Cerebellum - Smith et al. network map 5(20) | Cerebellum - Smith et al. network map 5(20) | TRUE | d40-Signal-IC |
| **19** | 0,480394486 | 3 | 0,460439478 | 2 | 0,691060916 | 3 | Visual-3 Smith et al.network map 3(20) | Visual-3 Smith et al.network map 3(20) | TRUE | d40-Signal-IC |
| **24** | 0,35290961 | 10 | 0,403607153 | 9 | 0,278153038 | 9 | Frontoparietal – Smith et al. network map 10(20) | Frontoparietal - Smith et al. network map 9(20) | NOT TRUE | d40-Signal-IC |
| **29** | 0,323345803 | 7 | 0,584082193 | 17 | 0,36485525 | 7 | Auditory – Smith et al. network map 7(20) | Auditory – Smith et al. network map 7(20) | TRUE | d40-Signal-IC |
| **34** | 0,227521977 | 6 | 0,366240399 | 14 | 0,521687253 | 10 | Sensorimotor - Smith et al. network map 6(20) | Frontoparietal – Smith et al. network map 10(20) | NOT TRUE | d40-Signal-IC |
| **37** | 0,255345155 | 6 | 0,333049132 | 18 | 0,528885005 | 6 | Sensorimotor - Smith et al. network map 6(20) | Sensorimotor - Smith et al. network map 6(20) | TRUE | d40-Signal-IC |
| **40** | 0,210827875 | 8 | 0,396043679 | 14 | 0,521687253 | 10 | Executive control – Smith et al. network map 8(20) | Frontoparietal – Smith et al. network map 10(20) | NOT TRUE | d40-Signal-IC |
| **46** | 0,112232374 | 3 | 0,216071904 | 27 | 0,147540039 | 8 | Visual-3 Smith et al.network map 3(20) | Executive control – Smith et al. network map 8(20) | NOT TRUE | d40-Signal-IC |
| **47** | 0,074946394 | 9 | 0,354927727 | 15 | 0,084007096 | 3 | Frontoparietal - Smith et al. network map 9(20) | Visual-3 Smith et al.network map 3(20) | NOT TRUE | d40-Signal-IC |
| **50** | 0,148257229 | 6 | 0,198954545 | 23 | 0,493164908 | 5 | Sensorimotor - Smith et al. network map 6(20) | Cerebellum - Smith et al. network map 5(20) | NOT TRUE | d40-Signal-IC |
| **4** | 0,116421896 | 8 | 0,42447506 | 24 | 0,259262132 | 6 | Executive control – Smith et al. network map 8(20) | Sensorimotor - Smith et al. network map 6(20) |  | d40-Noise-IC |
| **16** | 0,279642651 | 8 | 0,389417395 | 26 | 0,266777118 | 8 | Executive control – Smith et al. network map 8(20) | Executive control – Smith et al. network map 8(20) |  | d40-Noise-IC |
| **27** | 0,070886248 | 4 | 0,292004103 | 34 | 0,226955534 | 5 | DMN - Smith et al. network map 4(20) | Cerebellum - Smith et al. network map 5(20) |  | d40-Noise-IC |
| **32** | 0,154088236 | 6 | 0,46767428 | 24 | 0,259262132 | 6 | Sensorimotor - Smith et al. network map 6(20) | Sensorimotor - Smith et al. network map 6(20) |  | d40-Noise-IC |
| **33** | 0,084846783 | 3 | 0,663243361 | 31 | 0,082545128 | 5 | Visual-3 Smith et al.network map 3(20) | Cerebellum - Smith et al. network map 5(20) |  | d40-Noise-IC |
| **35** | 0,080869479 | 7 | 0,785319408 | 19 | 0,138398598 | 6 | Auditory – Smith et al. network map 7(20) | Sensorimotor - Smith et al. network map 6(20) |  | d40-Noise-IC |
| **38** | 0,095769404 | 7 | 0,269359039 | 29 | 0,155891964 | 7 | Auditory – Smith et al. network map 7(20) | Auditory – Smith et al. network map 7(20) |  | d40-Noise-IC |
| **39** | 0,399128131 | 2 | 0,569165624 | 37 | 0,281473292 | 2 | Visual-2 Smith et al.network map 2(20) | Visual-2 Smith et al.network map 2(20) |  | d40-Noise-IC |
| **41** | 0,081917331 | 8 | 0,611747411 | 38 | 0,102131758 | 5 | Executive control – Smith et al. network map 8(20) | Cerebellum - Smith et al. network map 5(20) |  | d40-Noise-IC |
| **42** | 0,074269373 | 3 | 0,533670014 | 30 | 0,124717111 | 10 | Visual-3 Smith et al.network map 3(20) | Frontoparietal – Smith et al. network map 10(20) |  | d40-Noise-IC |
| **43** | 0,085288209 | 5 | 0,436674402 | 29 | 0,155891964 | 7 | Cerebellum - Smith et al. network map 5(20) | Auditory – Smith et al. network map 7(20) |  | d40-Noise-IC |
| **44** | 0,233634402 | 7 | 0,433210654 | 36 | 0,139718315 | 7 | Auditory – Smith et al. network map 7(20) | Auditory – Smith et al. network map 7(20) |  | d40-Noise-IC |
| **45** | 0,149793087 | 8 | 0,528116099 | 26 | 0,266777118 | 8 | Executive control – Smith et al. network map 8(20) | Executive control – Smith et al. network map 8(20) |  | d40-Noise-IC |
| **48** | 0,179411564 | 5 | 0,3236931 | 39 | 0,138809485 | 5 | Cerebellum - Smith et al. network map 5(20) | Cerebellum - Smith et al. network map 5(20) |  | d40-Noise-IC |
| **49** | 0,147329061 | 6 | 0,271689292 | 24 | 0,259262132 | 6 | Sensorimotor - Smith et al. network map 6(20) | Sensorimotor - Smith et al. network map 6(20) |  | d40-Noise-IC |

**References**

1. Krupp LB, LaRocca NG, Muir-Nash J, Steinberg AD. The fatigue severity scale. Application to patients with multiple sclerosis and systemic lupus erythematosus. Arch Neurol. 1989;46(10):1121-3.

2. Penner IK, Paul F. Fatigue as a symptom or comorbidity of neurological diseases. Nature reviews Neurology. 2017.

3. Smith A. Symbol Digit Modalities Test (SDMT). Manual (Revised). Los Angeles: Western Psychological Services. 1982.

4. Skorve E, Lundervold AJ, Torkildsen O, Myhr KM. The Norwegian translation of the brief international cognitive assessment for multiple sclerosis (BICAMS). Multiple sclerosis and related disorders. 2019;36:101408.

5. Delis DC, Kaplan, E., & Kramer, J. H. Delis-Kaplan executive function system: Examiner’s manual. San Antonio TTPC, editor: San Antonio, TX: The Psychological Corporation.; 2001.

6. Delis DC, Kaplan, E., Kramer, J. H., & Ober, B. A. . California Verbal Learning Test - Second Edition - Adult Version (CVLT-II). 2000.

7. Eijlers AJC, van Geest Q, Dekker I, Steenwijk MD, Meijer KA, Hulst HE, et al. Predicting cognitive decline in multiple sclerosis: a 5-year follow-up study. Brain. 2018;141(9):2605-18.

8. Dale AM, Fischl B, Sereno MI. Cortical surface-based analysis. I. Segmentation and surface reconstruction. Neuroimage. 1999;9(2):179-94.

9. Battaglini M, Jenkinson M, De Stefano N. Evaluating and reducing the impact of white matter lesions on brain volume measurements. Human brain mapping. 2012;33(9):2062-71.

10. Reuter M, Schmansky NJ, Rosas HD, Fischl B. Within-subject template estimation for unbiased longitudinal image analysis. Neuroimage. 2012;61(4):1402-18.

11. Damangir S, Manzouri A, Oppedal K, Carlsson S, Firbank MJ, Sonnesyn H, et al. Multispectral MRI segmentation of age related white matter changes using a cascade of support vector machines. J Neurol Sci. 2012;322(1-2):211-6.

12. Brewer JB. Fully-automated volumetric MRI with normative ranges: translation to clinical practice. Behav Neurol. 2009;21(1):21-8.

13. W. Luo KL, A. M. Ulug, J. Albright, S. Magda, R. Haxton, C. Airriess. LesionQuant Performance Evaluation

Accuracy and Reproducibility (white paper): Cortechs Labs; 2017.

14. Smith SM, Jenkinson M, Woolrich MW, Beckmann CF, Behrens TE, Johansen-Berg H, et al. Advances in functional and structural MR image analysis and implementation as FSL. Neuroimage. 2004;23 Suppl 1:S208-19.

15. Jenkinson M, Bannister P, Brady M, Smith S. Improved optimization for the robust and accurate linear registration and motion correction of brain images. Neuroimage. 2002;17(2):825-41.

16. Smith SM. Fast robust automated brain extraction. Human brain mapping. 2002;17(3):143-55.

17. Smith SM, Brady JM. SUSAN—A New Approach to Low Level Image Processing. International Journal of Computer Vision. 1997;23(1):45-78.

18. Beckmann CF, DeLuca M, Devlin JT, Smith SM. Investigations into resting-state connectivity using independent component analysis. Philos Trans R Soc Lond B Biol Sci. 2005;360(1457):1001-13.

19. Griffanti L, Salimi-Khorshidi G, Beckmann CF, Auerbach EJ, Douaud G, Sexton CE, et al. ICA-based artefact removal and accelerated fMRI acquisition for improved resting state network imaging. Neuroimage. 2014;95:232-47.

20. Pruim RH, Mennes M, van Rooij D, Llera A, Buitelaar JK, Beckmann CF. ICA-AROMA: A robust ICA-based strategy for removing motion artifacts from fMRI data. Neuroimage. 2015;112:267-77.
